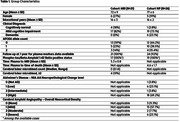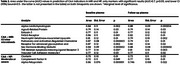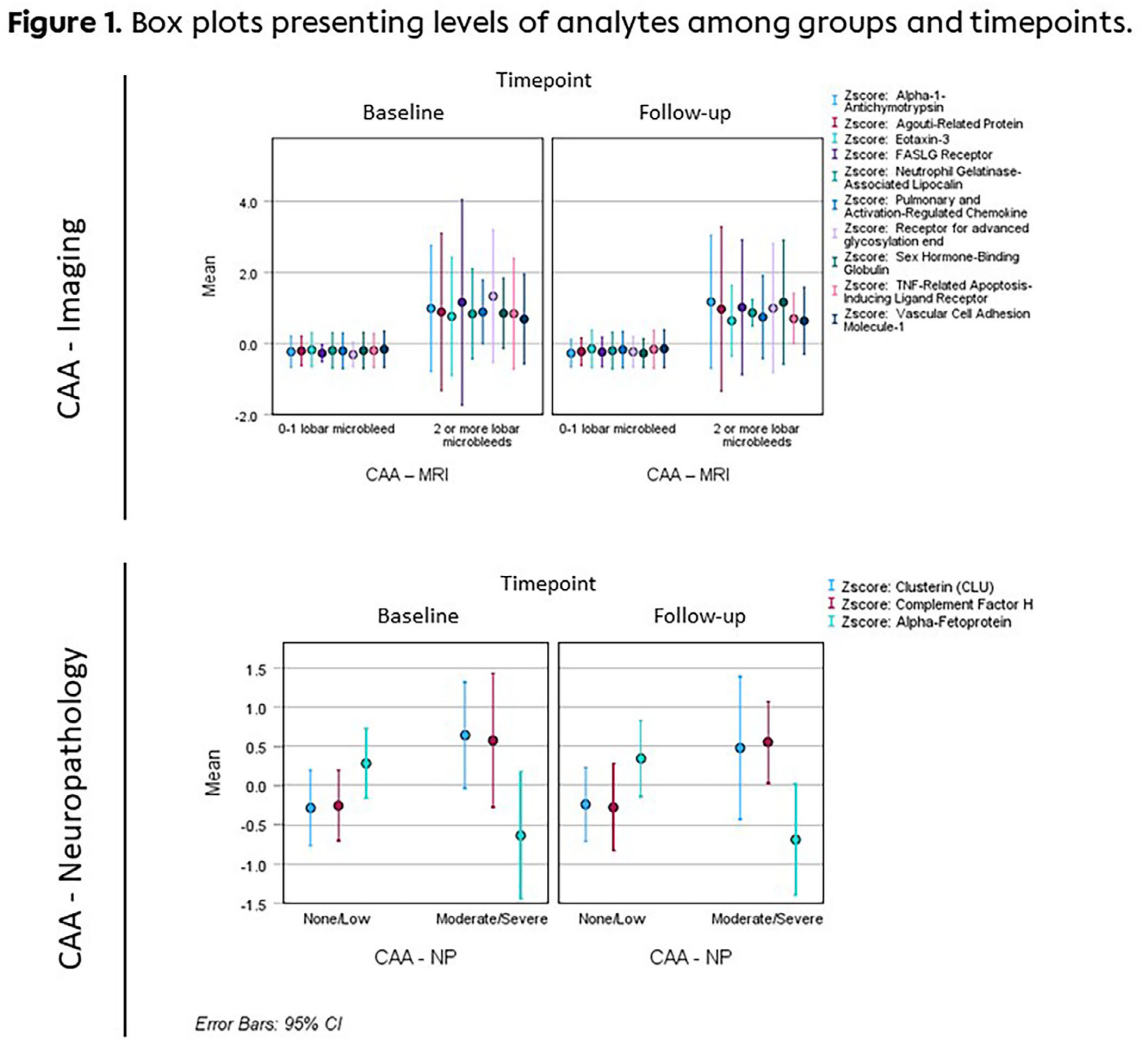# An explorative analyses of in vivo plasma marker alterations in relation to imaging and neuropathological indicators of cerebral amyloid angiopathy

**DOI:** 10.1002/alz70856_105585

**Published:** 2026-01-09

**Authors:** Ersin Ersoezlue, Julian Hellmann‐Regen

**Affiliations:** ^1^ Department of Psychiatry and Neurosciences, Charité Berlin University Medicine, Berlin, Berlin, Germany; ^2^ Experimental and Clinical Research Center (ECRC), Campus Berlin Buch, Charité Berlin University Medicine, Berlin, Berlin, Germany; ^3^ Department of Psychiatry and Neurosciences, Charité Universitätsmedizin Berlin, Berlin, Germany; ^4^ German Center for Neurodegenerative Diseases (DZNE), Berlin, Germany

## Abstract

**Background:**

Alzheimer‘s disease (AD) is one of the most prevalent causes of dementia, while concomitant diseases such as cerebral amyloid angiopathy (CAA) has a substantial impact on clinical trajectories and therapy, i.e. risk factor for imaging abnormalities under anti‐amyloid antibodies. As there are no established biomarkers to identify individual with CAA, we aim to explore potential plasma biomarkers for mechanisms related to CAA in participants in continuum of AD.

**Method:**

We included a total of 47 participants from the AD Neuroimaging Initiative study with available plasma biomarkers from a multiplex immunoassay panel (*n* = 145 analytes from “Biomarkers Consortium MRM data”, consisting of proteins related to cancer, cardiovascular disease, metabolic disorders, inflammation, and AD). We stratified the cohort into participants with either T2*‐GRE magnetic resonance images (MRI) (*n* = 21) at baseline or postmortem neuropathological assessment (*n* = 26). The numbers of definite lobar microbleeds were obtained from central visual readings (Mayo Clinic, Jack Lab), while central neuropathological severity scales for AD (AD neuropathologic change) and CAA (overall neocortical amyloid angiopathy) were included. We defined CAA status as at least two lobar microbleeds in orientation to the Boston criteria and at least moderate density in neuropathology. Plasma analytes were measured twice with a one‐year time difference with a maximum of 6.6 years prior to either first MRI or time of death. Non‐parametric receiver operating characteristic curves and area under the curve (AUC) values of analytes in differentiation of CAA status.

**Result:**

In both cohorts with imaging and NP data, most of the participants exhibited cognitive symptoms and revealed in vivo or neuropathological changes regarding AD (Table‐1). Using the imaging, various markers related to inflammation, lipid metabolism, cell adhesion, and sex steroids are found to show a constant increase in CAA (Table‐2, Figure 1). Moreover, we identified increases in Clusterin and Complement Factor H levels as well as reduced Alpha‐Fetoprotein, characterizing the neuropathological definition of CAA (Table‐2, Figure 1).

**Conclusion:**

Using both ante‐mortem and post‐mortem indicators of CAA, several candidate plasma biomarkers of CAA have been found, whereas replications in bigger samples with multiple measurements are crucial to address confounder factors and temporal relationships.